# The Redox Status of Cancer Cells Supports Mechanisms behind the Warburg Effect

**DOI:** 10.3390/metabo6040033

**Published:** 2016-10-03

**Authors:** Jorgelindo da Veiga Moreira, Minoo Hamraz, Mohammad Abolhassani, Erwan Bigan, Sabine Pérès, Loïc Paulevé, Marcel Levy Nogueira, Jean-Marc Steyaert, Laurent Schwartz

**Affiliations:** 1 Laboratoire d’Informatique de l’Ecole Polytechnique—Unité Mixte de Recherche 7161, Ecole Polytechnique, Palaiseau 91128, France; bigan.erwan@orange.fr (E.B.); marcel.levy@psl.aphp.fr (M.L.N.); jean-marc.steyaert@polytechnique.edu (J.-M.S.); 2 Imen Pharmed Iranian Company (IPI), Tehran 1514633711, Iran; mhamraz@ipiranian.com; 3 Nosco Pharmaceuticals, Paris 75015, France; mabolhas@noscopharm.fr; 4 Laboratoire de Recherche en Informatique (LRI), Université Paris-Sud, Centre National de la Recherche Scientifique Unité Mixte de Recherche 8623, Orsay 91190, France; sabine.peres@lri.fr (S.P.); loic.pauleve@lri.fr (L.P.); 5 MaIAGE, INRA, Université Paris-Saclay, Jouy-en-Josas 78350, France; 6 Institute of Translationnal Neurosciences (IHU-A-Institut du Cerveau et de la Moelle Epinière), Pitié Salpêtrière Hospital, Paris 75013, France

**Keywords:** redox oscillators, Warburg effect, cancer disease, mitochondria, central carbon metabolism

## Abstract

To better understand the energetic status of proliferating cells, we have measured the intracellular pH (pHi) and concentrations of key metabolites, such as adenosine triphosphate (ATP), nicotinamide adenine dinucleotide (NAD), and nicotinamide adenine dinucleotide phosphate (NADP) in normal and cancer cells, extracted from fresh human colon tissues. Cells were sorted by elutriation and segregated in different phases of the cell cycle (G0/G1/S/G2/M) in order to study their redox (NAD, NADP) and bioenergetic (ATP, pHi) status. Our results show that the average ATP concentration over the cell cycle is higher and the pHi is globally more acidic in normal proliferating cells. The NAD^+^/NADH and NADP^+^/NADPH redox ratios are, respectively, five times and ten times higher in cancer cells compared to the normal cell population. These energetic differences in normal and cancer cells may explain the well-described mechanisms behind the Warburg effect. Oscillations in ATP concentration, pHi, NAD^+^/NADH, and NADP^+^/NADPH ratios over one cell cycle are reported and the hypothesis addressed. We also investigated the mitochondrial membrane potential (MMP) of human and mice normal and cancer cell lines. A drastic decrease of the MMP is reported in cancer cell lines compared to their normal counterparts. Altogether, these results strongly support the high throughput aerobic glycolysis, or Warburg effect, observed in cancer cells.

## 1. Introduction

**Figure 1:**
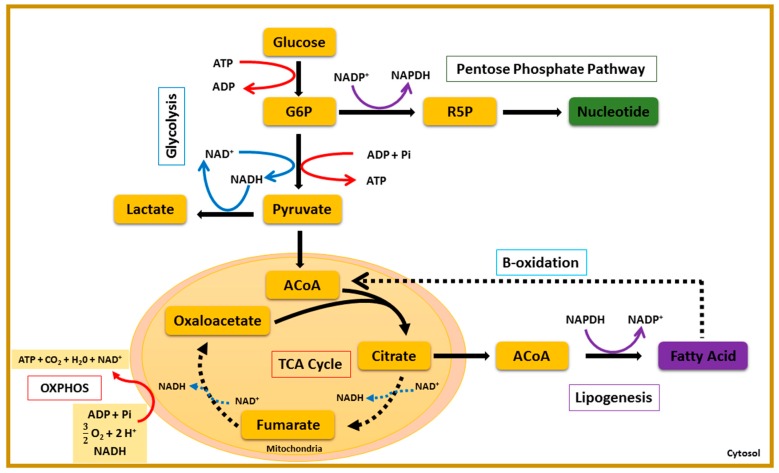
The central carbon metabolism (CCM). The CCM combines enzymatic reactions that convert carbon sources into biomass precursors. This figure shows the five main pathways forming the CCM: glycolysis, the pentose phosphate pathway, the tricarboxylic acid cycle (TCA), lipogenesis, and beta-oxidation. Two opposite metabolic demands are at the core of the CCM: anabolic reactions, which consist in biomass synthesis, and catabolic reactions, leading to the breakdown of macromolecules for energetic use. These two aspects of cell metabolism are managed by biochemical oscillators, including redox couples, such as nicotinamide adenine dinucleotide (NAD^+^/NADH) and nicotinamide adenine dinucleotide phosphate (NADP^+^/NADPH), and the universal energy carrier, adenine triphosphate (ATP/ADP). Transitions in CCM are reported to depend on these bio-oscillators. The NAD^+^/NADH ratio measures the glycolytic flux (glycolysis, PPP, and oxidative phosphorylation (OXPHOS). A high NADP^+^/NADPH ratio rewires glucose oxidation to the pentose phosphate pathway, whereas a low NADP^+^/NADPH ratio triggers lipogenesis. The ATP/(ADP + Pi) ratio senses the metabolic state of the cell and may lead to metabolic switches in the CCM.

The central carbon metabolism (CCM) is a metabolic blueprint shared by almost all living organisms. It consists of a complex series of enzymatic reactions that convert carbon sources (carbohydrates, proteins, lipids) into biomass and energy [1]. The core of the CCM is composed of glycolysis, the pentose phosphate pathway (PPP), the tricarboxylic acid cycle (TCA), and the fatty-acid pathway (Figure 1). The shift either from oxidative catabolism (energy production) to reductive anabolism (biomass synthesis), or from anabolism to catabolism, appears to be controlled by bio-oscillators, playing the role of co-enzymes [2]. These bio-oscillators include reductive and oxidative (redox) couples, such as nicotinamide adenine dinucleotide (NAD^+^/NADH) and nicotinamide adenine dinucleotide phosphate (NADP^+^/NADPH), the universal energy carrier, adenosine triphosphate (ATP), the transmembrane potential (TMP), and, finallyeast, the intracellular pH (pHi). The dynamics of these internal biological rhythms are shown to oscillate when eukaryotic cells enter proliferation [2,3,4]. These orchestrated oscillations ensure genome duplication and cell membrane synthesis prior to cell division [5,6].

The eukaryotic cell cycle has been shown to be a redox cycle [7,8,9,10]. When resting cells are committed to division, it enters the first growth phase (G1). Then follows genome duplication in the S phase and the second growth phase (G2), prior to mitosis (M). As opposed to proliferating cells, differentiated cells have a basal oxidative metabolism. Pyruvate converted from glucose or acetyl-CoA obtained from beta-oxidation of fatty acids are degraded by the TCA cycle (Figure 1) [11]. The oxidative phosphorylation of acetyl-CoA into mitochondria yields large amounts of ATP and releases carbon dioxide and water as waste products [2]. Thus, contrary to differentiated cells, rapidly proliferating cells, such as stem cells or cancer cells, use the aerobic glycolysis for ATP synthesis, even in the presence of oxygen. This is known as the “Warburg effect”, in reference to the Nobel laureate Otto Heinrich Warburg who first reported the high glycolytic flux in proliferating tumors [12,13,14]. In G1 of the cell cycle, the shift from pyruvate to lactate may be conditioned by the NAD^+^/NADH ratio and is thought to support the high glycolytic demand in cancer cells [5,12]. Thus, the Warburg effect may also be characterized by a high NAD^+^/NADH ratio. In the S phase, the CCM may shift to the PPP, the main route to nucleotide synthesis [4,5]. High metabolic flux through the PPP supplies enough NADPH to protect proliferating cells against oxidative stresses and triggers lipid synthesis in G2 [15,16,17]. Moreover, mitochondria activity is key to cell decision-making and cell cycle progression [18]. These organelles were first considered as a mere “powerhouse” of the eukaryotic cell, before the pioneering studies that deciphered their key role in such processes as cell development, cell survival, cell division, and cell death [18]. Regarding cell division, investigations support and highlight the idea of intertwined relationships between morphological changes in mitochondria and their signaling activity [18,19,20]. Subsequently, it has been demonstrated that energetic transitions occurring in the cell cycle are intrinsically related to mitochondrial ability to sense state parameters, such as pHi and the ATP/ADP ratio [21,22,23].

These measurements of pHi, ATP/ADP, and redox species are reported in several species, in different environmental conditions, but lack consistencies (pHi variations are contradictory [3,4,22]) and comprehensive overview of bio-oscillators variation (in yeast or human cancer cells [4,11,22]). Therefore, there is a need for a comprehensive analysis of changes in pHi, ATP/ADP, NAD^+^/NADH, and NADP^+^/NADPH during the proliferating cell cycle for healthy and cancerous human cells. Such analysis is a first step towards a holistic understanding of the coupling between electrochemistry and metabolism, and could ultimately lead to a better understanding of the principles operating current metabolic therapies and to identify new ones [24]. In this work, we studied the redox and energetic variations in freshly isolated normal and cancer cells extracted from human patients and controls (*n* = 8).

## 2. Results

**Figure 2:**
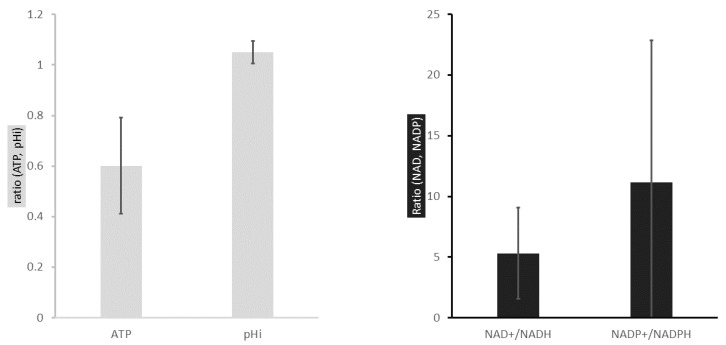
Comparison of the energetic status of normal and cancer cells. These histograms represent the average of ATP, pHi, NAD^+^/NADH, NADP^+^/NADPH cancer/normal ratios over the cell cycle. Raw data for all experiments are reported in the Appendix Section. (**A**) In normal cells, the average ATP concentration over the cell cycle is higher compared to the cancer cell population (ratio = 0.60 ± 0.19). The intracellular pH (pHi) of cancer cells is slightly higher than normal cells (ratio = 1.05 ± 0.04); and (**B**) the redox ratios (NAD^+^/NADH and NADP^+^/NADPH) are five and ten time higher in cancer cells compared to the normal cell population (5.31 ± 3.76 and 11.15 ± 11.71, respectively).

**Figure 3:**
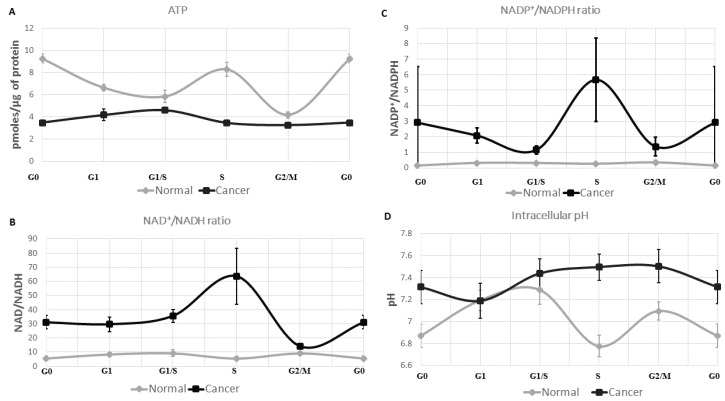
Redox signatures of normal and cancer cells during cell cycle progression. (**A**) In normal cells, the ATP concentration oscillates during the cell cycle: it is high in G0 (9.25 ± 0.40 pmoles/µg of protein) and S (8.29 ± 0.62 pmoles/µg of protein) and lower in G1/S (5.85 ± 0.57 pmoles/µg of protein) and G2/M (4.18 ± 0.29 pmoles/µg of protein). In cancer cells, ATP concentration is about twice lower in G0 (3.49 ± 0.25 pmoles/µg of protein), slightly increases from G0 to G1/S (4.6 ± 0.19 pmoles/µg of protein), and decreases from G1/S to G2/M (3.25 ± 0.11 pmoles/µg of protein); (**B**) in normal cells, the NAD^+^/NADH ratio oscillates between 5 and 10. For the cancerous cell line, the ratio increases from G0 (31.03 ± 1.54) and reaches a maximum value of 55 in S. Then, it declines at the G2/M transition (14); (**C**) the NADP^+^/NADPH ratio slightly increases from G0 (0.13 ± 0.05) to G1 (0.32 ± 0.04) in the normal cell population and stays stable. For the cancer cell population, a major peak is observed in S (5.68 ± 2.68); and (**D**) the intracellular pH (pHi) of cancer cells is alkaline and globally more acidic for normal cells. It oscillates from G0 (pH = 6.87 ± 0.10) to G1/S (pH = 7.29 ± 0.13), and then shows a marked decrease in S (pH = 6.78 ± 0.10). The cancerous cell population loses the pHi drop in the S phase and is much more alkaline (pH = 7.50 ± 0.12).

Human normal and cancer cells have been extracted from their respective tissues and isolated in five populations by the method of elutriation. This segregation, based on cell density, resulted in suspensions of cells in G0, G1, G1/S transition, S and G2/M transition phase of the cell cycle. By applying fluorometric measurements in each cell suspension, both in the normal and cancerous population, we managed to quantify the amount of ATP, NAD^+^, NADH, NADP^+^, NADPH, as well as the intracellular pH (pHi). Figure 2 and Figure 3 show experimental results obtained from eight patients (*n* = 8) for both the normal and the cancerous region of the colon. 

### 2.1. ATP Concentration Is Reduced in Colon Cancer Cells

The average ATP concentration (averaging over the various cell cycle phases) is about twice lower in colon cancer cells than in normal cells (Figure 2). This low energetic state characterizing cancer cells is already known from the literature where Otto Warburg first reported that rapidly-proliferating cells preferentially use the aerobic glycolysis/fermentation for ATP synthesis instead of oxidative phosphorylation [12,13,14]. Nevertheless, much less is known about the ATP concentration at the scale of one cell cycle. Thus, we report an oscillatory ATP concentration through all phases of a normal cell cycle (Figure 3A). Its concentration is found to be higher in G0 (9.25 ± 0.40 pmoles/µg of protein). Then, follows a decrease in G1 (6.63 ± 0.30 pmoles/µg of protein), and reaches a value of 5.85 ± 0.57 pmoles/µg of protein in G1/S. The ATP concentration increases from the G1/S transition to the S (8.29 ± 0.62 pmoles/µg of protein) phase, prior to a drop at the G2/M transition (4.18 ± 0.29 pmoles/µg of protein). In the colon cancer cell population the oscillatory phenotype of ATP concentration observed in their normal counterpart is lost. It slightly increases from G0 (3.49 ± 0.25 pmoles/µg of protein) to G1/S (4.6 ± 0.19 pmoles/µg of protein) and decreases through the S phase, until reaching a lower value in G2/M (3.25 ± 0.11 pmoles/µg of protein). These results, reported at the scale of one cell cycle, may suggest a global shift in ATP turnover in the cancer cell population compared to normal cells. Using the same method we also investigated the reductive and the oxidative (redox) status of the colonic normal and cancer cells.

### 2.2. Colon Cancer Cells Have a Reductive Energetic Status

**Figure A1:**
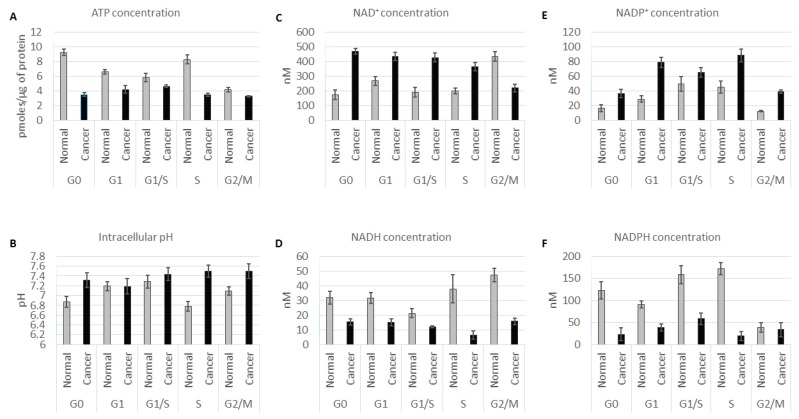
ATP, intracellular pH (pHi) and redox (NAD/NADH, NADP^+^/NADPH) concentrations and variations throughout colon normal and cancer cell cycle.

The nicotinamide adenine dinucleotide redox couple (NAD^+^/NADH) is a marker of catabolism, whereas its phosphorylated counterpart (NADP^+^/NADPH) is responsible for anabolism and antioxidant stress. Here, we aimed at describing the redox signature of the cancer cell population, compared to normal cells, both isolated from fresh human colon. These results show a divergent redox profile in cancer and normal cell populations. (Figure 2B and Figure 3B,C). Figure 2B shows the average of NAD^+^/NADH and NADP^+^/NADPH cancer/normal ratios for all phases of the cell cycle. NAD^+^/NADH ratio is higher in the cancer cell population (mean ratio = 5.31 ± 3.76) (Figure 2B). Considering these redox species individually, we note that NAD^+^ concentration is higher in cancer cells (Appendix Figure A1C), whereas its reductive counterpart has a lower concentration in the same cell population (Appendix Figure A1D). NAD^+^/NADH ratio oscillates throughout the cell cycle in these normal colon cell populations (Figure 3B). In normal cells, the catabolic ratio (NAD^+^/NADH) slightly increases from G0 (mean ratio = 5.59 ± 1.56) to the G1/S transition phase (mean ratio = 9.28 ± 2.54) prior to a drop in S (mean ratio = 5.44 ± 1.43), and another increasing phase in G2/M (mean ratio = 9.25 ± 1.09). The anabolic couple (NADP^+^ and NADPH) quantification is also reported. The NADP^+^/NADPH profiling ratio over the cell cycle is similar to the NAD^+^/NADH couple. The anabolic ratio is higher in cancer cells compared to the normal cell population (mean ratio = 11.15 ± 11.71) (Figure 2B). Thus, the cancer cell population shows anabolic ratio variations with more pronounced amplitudes (Figure 3C). It decreases from G0 (mean ratio = 2.91 ± 3.60) to G1 (mean ratio = 2.08 ± 0.48) and the G1/S transition phase (mean ratio = 1.16 ± 0.27). A major spike on the anabolic ratio is observed in S (mean ratio = 5.68 ± 2.68) followed by a second drop in G2/M (mean ratio 1.37 ± 0.60). An increased NADP^+^/NADPH ratio in cancer cells compared to normal ones may be related to the increased demand for nucleotide synthesis, lipogenesis, or/and reduction in oxidative stress. This is consistent with higher NADP^+^ and a decreased NADPH concentrations reported in cancer cells (Appendix Figure A1E,F). All taken together, these results are consistent with higher metabolic fluxes reported in the cancer population where a high NAD^+^/NADH ratio may sustain the aerobic glycolysis and an elevated NADP^+^/NADPH ratio would meet the anabolic demand for building blocks synthesis. Nevertheless, additional experiments, such as lactate quantification and oxidative stress measurements, are needed to fully confirm these assumptions.

### 2.3. The Intracellular pH of Cancer Cells Is Alkaline

In normal cell population, the pHi is slightly acidic, whereas the cancer cell population has an alkaline pHi (mean ratio = 1.05 ± 0.04) (Figure 2A). Thus, the pHi exhibits an oscillatory phenotype at the scale of one normal cell cycle, contrary to the cancer cell population where the pHi oscillation is reduced and plateaued at an alkaline value (Figure 3D). More precisely, normal cells have an acidic pHi in G0 (pH = 6.87 ± 0.10), which increases through G1 (pH = 7.19 ± 0.09) and G1/S G0 (pH = 7.29 ± 0.13). The intracellular pH drops in the S phase (pH = 6.78 ± 0.10). Then, it increases from the S phase to the G2/M transition (pH = 7.09 ± 0.08), prior to another drop during mitosis. These observations are in line with data from the literature [25,26].

### 2.4. Reduced Mitochondrial Membrane Potential in Immortal Cancer Cell Lines

Here, the goal was to depict possible correlations between alkaline pHi, low ATP concentration, the reductive metabolism (increased redox ratios), and lower mitochondria activity already hypothesized in cancer cells [13,14]. Indeed, mitochondria is often considered as the “powerhouse” of the cell since ATP is synthesized through the oxidative phosphorylation. Misregulations in this organelle may lead to cell death or metabolic diseases, such as cancer [12,19,22,27,28,29]. We did not manage to carry out these experiments in colon cells for lack of fresh colon cancer cells from patients. Instead, we used well-known human and mouse cell lines. Results are reported in Appendix Figuer A2A. What is clear from these results is the low mitochondrial membrane potential (MMP) reported in cancer cell lines compared to the normal primary cells. Thus, decreased MMP in cancer cells may explain the low ATP concentration observed in colon cancer cells, as well as in cancer cell lines.

## 3. Discussion

One of the main metabolic divergences between quiescent and proliferating cells is the pathway responsible for adenosine triphosphate (ATP) synthesis (Figure 1). In quiescent cells, ATP is synthesized during the oxidative phosphorylation (OXPHOS). Proliferating cells, such as stem cells, preferentially use the fermentative pathway for ATP synthesis in a “transient high throughput” manner, defined as the Warburg effect [13,14,25]. In the same manner as normal proliferating cells, cancer cells use the aerobic glycolysis (Warburg effect) for ATP synthesis in a “sustained high throughput” manner [30]. Here we hypothesized that the sustained fermentative character of cancer cell population may be correlated with shifts in redox potential and poor mitochondrial activity. As such, we quantified redox couples (NAD^+^/NADH, NADP^+^/NADPH) and ATP concentration at different phases of the cell cycle (G0, G1, G1/S, S, and G2/M) in normal and cancer colon cell populations. We also quantified the intracellular pH (pHi), which is a marker of high metabolic activity and measured the mitochondrial membrane potential (MMP) in the cell lines. These results bring a comprehensive understanding which needs additional study to fully support our hypothesis: we confirmed the general assumption of less effective ATP turnover in cancer cells. The redox ratios NAD^+^/NADH and NADP^+^/NADPH found in cancer cells support an enhanced glycolytic demand for sustained cell proliferation. Although the high NADP^+^/NADPH ratio in cancer cells is consistent with nucleotide demand for biomass synthesis, such a higher ratio may also imply a lower rate of synthesis for fatty acids, which are required for membrane synthesis. While a lower membrane synthesis may, at first sight, appear incompatible with a higher rate of biomass synthesis, such an incompatibility might actually be resolved by a change in cell shape. Moreover, both alkaline pHi and low mitochondrial membrane potential (MMP) in cancer cells may explain the Warburg effect. Different hypotheses could be addressed. Considering the generation of ATP by the movement of hydrogen ions across the inner mitochondrial membrane during cellular respiration, one would conclude that alkaline pHi may lead to a reduced ATP synthesis. Indeed, reduced oxidative phosphorylation and lower CO_2_ release may lead to less cytosolic acidification. Figure 3 showed ATP concentration, NAD^+^/NADH, NADP+/NADPH ratios, and pHi oscillation in normal and cancer colon cell populations (*n* = 8) throughout the cell cycle. ATP concentration is shown to be low in cancer cells compared to normal ones. On the contrary, the redox couple ratios (NAD^+^/NADH, NADP^+^/NADPH) are both very high in cancer cell populations. Indeed, a high catabolic ratio (NAD^+^/NADH) showed in Figure 2B and Figure 3B, especially in the S phase for the cancer cell population, which may explain a high glycolytic flux for building blocks synthesis [12]. Similarly, the anabolic ratio (NADP^+^/NADPH) is found to be high in cancer cells. Thus, lower ATP concentration in cancer cells, compared to normal cells and the hybrid metabolism (high catabolism and anabolism) materialized by both high NAD^+^/NADH and NADP^+^/NADPH ratios, may explain the already reported metabolic advantage of cancer cells [31]. We also tried to capture the intracellular pH (pHi) dynamics in both normal and cancer colon cell population. Indeed, as described above, the pHi of normal proliferating cells is globally acidic, whereas it is alkaline in cancer cells. Moreover, the pHi exhibits an oscillatory phenotype in normal cells and is flat in cancer cells. In order to explain these oscillations, additional experiments in pHi variation and a dynamic model of the central carbon metabolism is needed. Nevertheless, evidence has been reported [30], and recent findings highlighted, the relationship between pHi, chromatin remodeling, and the transcriptional activity in proliferating cells. Indeed, McBrian (2013), showed that pHi regulates histone acetylation which, in turn, conditions gene transcription [32,33,34]. He showed that alkaline pHi induces histone acetylation, whereas acidic pHi is linked to a deacetylated histone and, by extension, to a silenced transcription. Interestingly, deacetylation of histone is known to be a NAD-dependent mechanism [35]. Thus, it is likely that pHi and NAD^+^ regulate cell transcriptional activity and cell fate decision. Puysségur (1985) showed the existence of a pHi threshold of 7.2, which conditions entry into the S phase. Moreover, Aerts (1985) demonstrated that an increase in pHi results in both increased protein synthesis and DNA replication [26]. He reported the optimal protein and DNA synthesis being correlated with an alkaline pHi of 7.4. Busa [36] showed that pHi oscillations are master regulators of the embryonic cells’ decision-making to stay in dormancy or to enter the cell cycle [36]. He reported that an acidic pHi is linked to dormancy, whereas an alkaline pHi is a characteristic of rapidly proliferating cells—a hallmark of cancerous tissues. All of these data taken together, we propose a swaddling between the metabolic blueprint of the central carbon metabolism (CCM) (Figure 1) and the energetic demand for cell proliferation. These bio-oscillators, including ATP, pHi, and redox couples (NAD^+^/NADH, NADP^+^/NADPH) seem to coordinate switches in the CCM during normal cell proliferation. Misregulations on these balances could have a drastic impact on the integrity of the eukaryotic cell cycle, which could result in uncontrolled growth, such as in cancer. Results obtained with different normal and cancer cell lines reported lower MMP in cancer cells compared to normal cells (Appendix Figure A1A). To our knowledge, this is the first time experimental results have characterized the redox status of cancer cells by considering oscillations in the central carbon metabolism, where the energetic markers (ATP, pHi, and TMP) and, more importantly, the redox species (NAD^+^/NADH and NADP^+^/NADPH), may explain the Warburg effect mechanisms.

## 4. Materials and Methods

### 4.1. Cell Suspension Preparation and Centrifugal Elutriation

Normal and cancerous segments of 12 to 18 cm of the upper colon from eight 57–62 year-old female patients with tumors (adenocarcinoma) were obtained from the internal ward of the gastroenterology-surgical unit at the Teheran Medical Sciences University Imam Khomeini Hospital in Iran (Nosco Pharmaceuticals’ partnership, Paris, France). The epithelial and intraepithelial lymphocytes (IEL) were detached, as described previously [37]. The normal and cancerous colon segments from each patient were quickly made into a loop, inverted, and then incubated in a saline solution with 5 mM EDTA (Sigma-Aldrich, St. Louis, MO, USA) at 4 °C, while shaking gently to dislodge the colon crypts. The isolated crypts were then collected and incubated for 30 min at 37 °C in phosphate-buffered saline (PBS) solution without calcium containing 10% trypsin-EDTA (Gibco-Life Technologies, Cergy Pontoise, France). The cells were used for fresh culture. The isolated cell suspension of the colon epithelium (colonocytes) was distributed into 15-mL Falcon tubes containing 3 mL of cold fetal calf serum (Eurobio, Montpellier, France) and centrifuged for 5 min at 750 rpm. Percoll (Sigma-Aldrich, St. Louis, MO, USA) solutions (70% and 44%) were prepared for the isolation of human IEL and enterocytes. Up to 40 million epithelial cell-IEL suspensions were loaded per 15-mL tube by resuspending with 8 mL of 44% isotonic Percoll. This cell suspension was underplayed with 4 mL 70% isotonic Percoll. The gradients were centrifuged at 1500 rpm for 20 min (without breaks) at room temperature. Upon centrifugation, cells were collected in five fractions. Isolated cells of the epithelial cell layer of human colonocytes were centrifuged in a 50-mL Falcon tube for 5 min at 750 rpm and resuspended in 50 mL of MEM-alpha modification with l-glutamine medium (Sigma-Aldrich, St. Louis, MO, USA). After passing through the double chamber, the cell sample was transferred to the elutriation chamber with the elutriation buffer at a flow rate of 10 mL/min. A Beckmann Coulter centrifuge (Villepinte, France, model J-6M) with two JE-6B and E-10M rotors containing the 40 and 50-mL elutriation chamber and a Masterflex L/S digital pump (Cole-Parmer Instrument Co., Barrington, IL, USA) was used. Before each elutriation run, 200 mL of 70% ethanol was run though the elutriation chamber at about 60 mL/min (washing flow rate) to sterilize the system. This was sequentially followed by a wash with 500 mL of water, with 500 mL of PBS at approximately 60 mL/min and 2000 rpm to check for leaks and bubbles in the system. The loading/washing buffers are contained in the buffer reservoir connected to the Masterflex pump via a T-valve. After a pressure check, the fluid is reversed into a bubble chamber to avoid air bubbles in the rotor. Subsequently, the sample with the cell suspension and the elutriation buffer was forced into the elutriation chamber.

The separation of the cell suspension in the elutriation chamber was controlled virtually through the viewing point in the rotor. During the loading phase platelets and contaminating erythrocytes were already eluted from the elutriation chamber (fraction 1). Once all the cells were loaded and accumulated in the elutriation chamber (after approximately 10 min), the flow rate was increased to 20 mL/min at a constant rotor speed of 2000 rpm. The elutriated lymphoid cells were collected (fraction 2). Thereafter, the flow rate was increased to 40 mL/min (at a constant rotor speed of 2000 rpm) to elutriate the epithelial cell fraction (fraction 3, in three tubes of 45 mL each). At the end of the procedure, we obtained about 500 million epithelial cells that must be resuspended in 4 L of the medium. The median cell volumes for G1, S, and G2/M cells were respectively 620, 950, and 1260 µm^3^ and the corresponding rotor speeds (E-10M rotor containing the 50-mL elutriation chamber) for cell separation were 1160–1050 rpm, 1000–950 rpm, and 900–850 rpm, respectively. We started centrifugation at 2000 rpm and gradually reduced the speed against a 40 mL/min force of injection. Prolonged centrifugation permits separation of cells from these three categories and we collected five fractions of 12–15 million cells. For this study, informed consent was obtained from all subjects. All of the methods involving human subjects were performed in accordance with the standard practice procedures, regulations, and guidance, and were approved by the Teheran Imam Khomeini Hospital.

### 4.2. Fluorescence and Luminescence Measurements

All fluorescence and luminescence measurements have been made using a NOVOstar multifunctional microplate reader from Isogen Life Science (Utrecht, The Netherlands) and were analyzed using BMG Labtech software V1.30 R9 (Offenburg, Germany).

### 4.3. pHi Measurement

Intracellular calibration of the fluorescence response to total cytosolic pH indicators was performed using K^+^/H^+^ ionophore nigericin (Invitrogen, Cergy Pontoise, France), which causes equilibration of intracellular and extracellular pH in the presence of a depolarizing concentration of extracellular K^+^. After dissolving nigericin in 354 µL anhydrous Dimethyl Sulfoxide (DMSO) to make a 20 mM solution, it was mixed with 100 µL of Valinomycin (Sigma-Aldrich, St. Louis, MO, USA), used as an ionophore, to make a 1000× stock solution (10 mM each) for cell loading dye components. For cell acidification, we used the NH_4_^+^/NH_3_ (20 mmol/L) prepulse technique. We used the Cell Meter Fluorometric Intracellular pH (pHi) Assay KitTM (AAT Bioquest^®^, Sunnyvale, CA, USA). Cells were dispatched overnight in MEM alpha medium at 80,000 cells/well/100 µL for a 96-well plate. Stock solutions were made and the pH assay was run for 20 min by monitoring the fluorescence at Ex/Em = 490/535 nm (cut-off at 515 nm).

### 4.4. Reduced Mitochondrial Membrane Potential in Immortal Cancer Cell Lines

In all assays, we loaded the wells with 200,000 cells and the results were normalized using total protein concentrations in eight independent experiments (*n* = 8) corresponding to eight patients. All procedures followed the kit instructions: ENLITEN^®^ ATP Assay System (Promega FF2000; Charbonnières, France) for ATP quantification, NADP^+^/NADPH and NAD^+^/NADH assays were run using the NADP/NADPH-Glo Bioluminescent Assay kit (Promega G9082) and the NAD/NADH-Glo Bioluminescent Assay kit (Promega G9072), respectively. The assay of total intracellular (cytosol and organelles) ATP quantification, luciferase is used as the catalyzing enzyme of the ATP reaction with d-Luciferin. ATP being the limiting component of the reaction, the intensity of the emitted light is proportional to the amount of ATP. ATP extraction from cells is primordial and each step must limit ATP degradation. Briefly, ATP extraction is carried out with trichloroacetic acid reagent which releases ATP from the cell population and prevents ATP degradation by potential enzymes. After addition of the enzyme regent to the intracellular extract, light emission is detected by a luminometer at 560 nm. The assay of total intracellular (cytosol and organelles) NAD(P) and NAD(P)H were performed individually following the Promega protocol. For both couples, cells are lysed with dodecyltrimethyl ammonium bromide (DTAB) and treated to neutralize their counterparts. To measure the oxidized forms (NAD^+^ and NADP^+^) the extract is treated with 25 μL of 0.4 N HCl and heated at 60 °C for 15 min. Then follows a 10 min incubation at room temperature and the addition of 25 μL of Trizma^®^ (Sigma-Aldrich, St. Louis, MO, USA) base. NAD^+^ and NADP^+^ are measured using a NAD/NADH-Glo™ (Charbonnières, France) assay and NADP/NADPH-Glo™ assay, respectively. To quantify the reduced forms (NADH and NADPH), the extract is incubated at 60 °C for 15 min and then another 10 min incubation at room temperature. Fifty microliters of HCl/Trizma^®^ solution is added to the extract. Similarly to the oxidized forms, NADH and NADPH are measured using a NAD/NADH-Glo^™^ assay and a NADP/NADPH-Glo™ assay, respectively. The specificity of each assay (NAD or NADP) is related to the cycling enzymes used. In the presence of each species, a reductase reduces a proluciferin reductase to luciferin. The intensity of light detected by a luminometer is proportional to the amount of each metabolite.

## 5. Conclusions

The metabolic description of the cancer phenotype introduced by Otto Warburg has been supported by numerous studies focusing on the fermentative aspect of it. Far fewer studies have examined the reductive and oxidative status of cancer cells in order to explain their metabolic reprogramming and the resulting hybrid metabolism where catabolism and anabolism occur at the same time. Consequently, we reported low ATP concentration, high NAD^+^/NADH and NADP^+^/NADPH ratios, and low mitochondrial membrane potential in cancer cells, compared to normal proliferating cells. These results give new perspectives on aerobic glycolysis, or the Warburg effect. Therapies should focus on improving the oxidative phosphorylation in cancer cells. Drugs such as dichloroacetate [38,39], hydroxycitrate, and lipoic acid [24,40] have been used to increase mitochondrial yield for ATP and decrease cancer growth in rodents and humans.

## Figures and Tables

**Figure A2:**
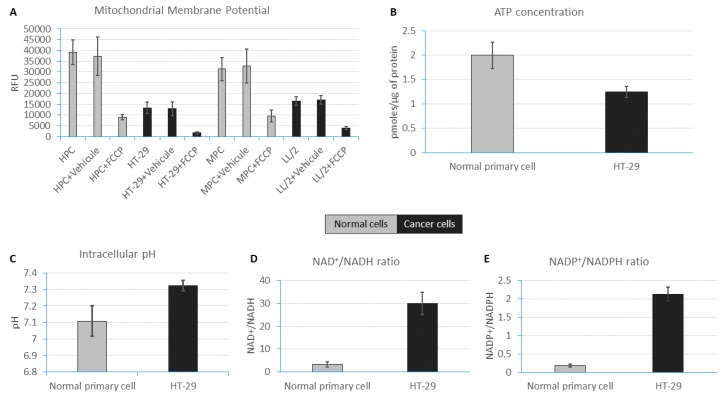
MMP, ATP, pHi and redox species concentrations in normal and cancer cell lines. (**A**) MMP are measured in human and mouse normal and cancer cell lines. FCCP is a compound that carries protons across the IMM and dissipates the electrochemical gradient (membrane potential). In order to maintain the membrane potential, the mitochondria need to increase the flow of electrons and thus oxygen consumption. Optimizing the concentration of the uncoupler (FCCP) reveals the maximal respiration for the sample; (**B**) The ATP concentration is lower in HT-29 compared to normal primary cells; (**C**) The intracellular pH (pHi) is alkaline in HT-29; (**D**) The NAD^+^/NADH ratio is higher in HT-29 cancerous line compared to normal primary cells; (**E**) The NADP^+^/NADPH ratio is higher in HT-29 cancerous line. Abbreviations: MMP (mitochondrial membrane potential); FCCP (carbonyl cyanide-p-trifluoromethoxyphenylhydrazone); IMM (internal mitochondrial membrane); HPC (Hematopoietic progenitor cells), LL/2 (mouse Lewis lung cancer cell line); MPC (mouse normal pheochromocytoma cell line).

## References

[1] Noor E., Eden E., Milo R., Alon U. (2010). Central carbon metabolism as a minimal biochemical walk between precursors for biomass and energy. Mol. Cell.

[2] Da Veiga Moreira J., Peres S., Steyaert J.-M., Bigan E., Paulevé L., Nogueira M.L., Schwartz L. (2015). Cell cycle progression is regulated by intertwined redox oscillators. Theor. Biol. Med. Model..

[3] Tyson J.J. (2002). Biochemical oscillations. Computational Cell Biology.

[4] Yu F.X., Dai R.P., Goh S.R., Zheng L., Luo Y. (2009). Logic of a mammalian metabolic cycle: An oscillated NAD^+^/NADH redox signaling regulates coordinated histone expression and S-phase progression. Cell Cycle.

[5] Diaz-Moralli S., Tarrado-Castellarnau M., Miranda A., Cascante M. (2013). Targeting cell cycle regulation in cancer therapy. Pharmacol. Ther..

[6] Norbury C., Nurse P. (1992). Animal cell cycles and their control. Annu. Rev. Biochem..

[7] Nurse P. (2000). A long twentieth century of the cell cycle and beyond. Cell.

[8] Burhans W.C., Heintz N.H. (2009). The cell cycle is a redox cycle: Linking phase-specific targets to cell fate. Free Radic. Biol. Med..

[9] Chiu J., Dawes W. (2012). Redox control of cell proliferation. Trends Cell Biol..

[10] Menon S.G., Goswami P.C. (2007). A redox cycle within the cell cycle: Ring in the old with the new. Oncogene.

[11] Sarsour E.H., Kumar M.G., Chaudhuri L., Kalen A.L., Goswami P.C. (2009). Redox control of the cell cycle in health and disease. Antioxid. Redox Signal..

[12] DeBerardinis R.J., Lum J.J., Hatzivassiliou G., Thompson C.B. (2008). The biology of cancer: Metabolic reprogramming fuels cell growth and proliferation. Cell Metab..

[13] Warburg O. (1925). The metabolism of carcinoma cells. J. Cancer Res..

[14] Warburg O. (1956). On the origin of cancer cells. Science.

[15] Fabregat I., Vitorica J., Satrustegui J., Machado A. (1985). The pentose phosphate cycle is regulated by NADPH/NADP ratio in rat liver. Arch. Biochem. Biophys..

[16] Fabregat I., Revilla E., Machado A. (1987). Short-term control of the pentose phosphate cycle by insulin could be modulated by the NADPH/NADP ratio in rat adipocytes and hepatocytes. Biochem. Biophy. Res. Commun..

[17] Revilla E., Fabregat I., Santa-María C., Machado A. (1987). The NADPH-producing pathways (pentose phosphate and malic enzyme) are regulated by the NADPH consumption in rat mammary gland. Biochem. Int..

[18] McBride H.M., Neuspiel M., Wasiak S. (2006). Mitochondria: More than just a powerhouse. Curr. Biol..

[19] Scalettar B.A., Abney J.R., Hackenbrock C.R. (1991). Dynamics, structure, and function are coupled in the mitochondrial matrix. Proc. Natl. Acad. Sci. USA.

[20] Hackenbrock C.R. (1968). Ultrastructural bases for metabolically linked mechanical activity in mitochondria II. Electron transport-linked ultrastructural transformations in mitochondria. J. Cell Biol..

[21] Mitra K., Wunder C., Roysam B., Lin G., Lippincott-Schwartz J. (2009). A hyperfused mitochondrial state achieved at G1–S regulates cyclin E buildup and entry into S phase. Proc. Natl. Acad. Sci. USA.

[22] Christen R., Schackmann R.W., Shapiro B.M. (1983). Metabolism of sea urchin sperm. Interrelationships between intracellular pH, ATPase activity, and mitochondrial respiration. J. Biol. Chem..

[23] Jones R.G., Plas D.R., Kubek S., Buzzai M., Mu J., Xu Y., Birnbaum M.J., Thompson C.B. (2005). AMP-activated protein kinase induces a p53-dependent metabolic checkpoint. Mol. Cell.

[24] Schwartz L., Buhler L., Icard P., Lincet H., Steyaert J.M. (2014). Metabolic treatment of cancer: Intermediate results of a prospective case series. Anticancer Res..

[25] Pouyssegur J., Franchi A., L’allemain G., Paris S. (1985). Cytoplasmic pH, a key determinant of growth factor-induced DNA synthesis in quiescent fibroblasts. FEBS Lett..

[26] Aerts R.J., Durston A.J., Moolenaar W.H. (1985). Cytoplasmic pH and the regulation of the Dictyostelium cell cycle. Cell.

[27] Finkel T., Hwang P.M. (2009). The Krebs cycle meets the cell cycle: Mitochondria and the G1–S transition. Proc. Natl. Acad. Sci. USA.

[28] Westrate L.M., Sayfie A.D., Burgenske D.M., MacKeigan J.P. (2014). Persistent mitochondrial hyperfusion promotes G2/M accumulation and caspase-dependent cell death. PLoS ONE.

[29] Toyama E.Q., Herzig S., Courchet J., Lewis T.L., Losón O.C., Hellberg K., Young N.P., Chen H., Polleux F., Chan D.C. (2016). AMP-activated protein kinase mediates mitochondrial fission in response to energy stress. Science.

[30] Zhang S., Yang C., Yang Z., Zhang D., Ma X., Mills G., Liu Z. (2015). Homeostasis of redox status derived from glucose metabolic pathway could be the key to understanding the Warburg effect. Am. J. Cancer Res..

[31] Israël M., Schwartz L. (2011). The metabolic advantage of tumor cells. Mol. Cancer.

[32] McBrian M.A., Behbahan I.S., Ferrari R., Su T., Huang T.W., Li K., Hong C.S., Christofk H.R., Vogelauer M., Seligson D.B. (2013). Histone acetylation regulates intracellular pH. Mol. Cell.

[33] Kurdistani S.K. (2014). Chromatin: A capacitor of acetate for integrated regulation of gene expression and cell physiology. Curr. Opin. Genet. Dev..

[34] Kurdistani S.K., Grunstein M. (2003). Histone acetylation and deacetylation in yeast. Nat. Rev. Mol. Cell Biol..

[35] Imai S.I., Armstrong C.M., Kaeberlein M., Guarente L. (2000). Transcriptional silencing and longevity protein Sir2 is an NAD-dependent histone deacetylase. Nature.

[36] Busa W.B., Nuccitelli R. (1984). Metabolic regulation via intracellular pH. Am. J. Physiol..

[37] Abolhassani M., Aloulou N., Chaumette M.T., Aparicio T., Martin-Garcia N., Mansour H., Le Gouvello S., Delchier J.C., Sobhani I. (2008). Leptin receptor–related immune response in colorectal tumors: The role of colonocytes and interleukin-8. Cancer Res..

[38] Michelakis E.D., Abdulkarim B. (2010). Metabolic modulation of glioblastoma with dichloroacetate. Sci. Transl. Med..

[39] Kinnaird A., Michelakis E. (2012). 297 Dichloroacetate is a novel therapy for renal cell carcinoma. J. Urol..

[40] Kafara P., Icard P., Guillamin M., Schwartz L., Lincet H. (2015). Lipoic acid decreases Mcl-1, Bcl-x L and up regulates Bim on ovarian carcinoma cells leading to cell death. J. Ovarian Res..

